# A Retrospective Study on the Effectiveness of Ixekizumab After Treatment With Secukinumab for Patients With Active Psoriatic Arthritis

**DOI:** 10.1177/24755303211063841

**Published:** 2021-12-20

**Authors:** Saman Darabian, Maziar Badii, Jan P. Dutz, Jonathan Chan

**Affiliations:** 1 Faculty of Arts, University of British Columbia, Vancouver, BC, Canada; 2 Department of Medicine, University of British Columbia, Vancouver, BC, Canada; 3 Department of Dermatology and Skin Science, 8166 University of British Columbia, Vancouver BC, Canada

**Keywords:** psoriatic arthritis, DMARDs (biologic), ixekizumab, secukinumab

## Abstract

**Objectives:** This study aims to evaluate clinical responses in patients with active psoriatic arthritis who, despite secukinumab 300 mg subcutaneous monthly, are switched to ixekizumab 80 mg subcutaneous every four weeks. **Methods:** We conducted a chart review of adult patients with psoriatic arthritis treated at one clinical center. We identified all patients with active inflammatory arthritis who were switched from secukinumab to ixekizumab. Baseline demographics such as disease duration, age, gender, number of previous DMARDs, and previous time on secukinumab were collected. We collected clinical outcome data such as tender and swollen joint count, enthesitis based on SPARCC score, dactylitis, psoriasis severity, CRP, and BASDAI if axial involvement was present. **Results:** Eight of 10 patients were included in the analysis. Most patients were female, average age 62 years old, and had been on secukinumab for an average of 79 weeks. Twelve weeks following switch to ixekizumab, 6/8 had improvement in tender joint count, 6/8 improved in swollen joint count, 2/2 had resolution of enthesitis, 4/4 had resolution of dactylitis, 5/6 had improvement in psoriasis severity, 1 patient had absolute improvement of 2.3 in BASDAI, and 7/8 had improvement in the CRP level. **Conclusions:** Patients with active psoriatic arthritis despite treatment with secukinumab may still have a clinical response following treatment with another anti-IL17 agent. Larger studies will be required to confirm this finding, and studies which emphasize dactylitis and enthesitis outcomes will be needed as most patients did not have activity in these domains.

## Introduction

Psoriatic arthritis (PsA) is a chronic inflammatory disease associated with skin and nail psoriasis that can cause peripheral arthritis, spondylitis, enthesitis, and dactylitis. Interleukin (IL)-17 A is a cytokine that has been identified in the pathogenesis of PsA and two molecules, secukinumab and ixekizumab, which block IL17 A, have been shown in clinical trials to be effective for the treatment of this condition.^1-3^

To date, one randomized controlled trial has demonstrated efficacy when switching patients with axial spondyloarthritis who have an inadequate response to a TNF inhibitor to an IL17 A inhibitor.^4^ There have been no studies reporting the efficacy of ixekizumab in patients with active psoriatic arthritis and axial spondyloarthritis and a prior inadequate response to secukinumab. Consequently, clinicians may be hesitant to try a second IL17 A inhibitor and instead choose a therapy with an alternative mode of action. This study aims to assess the effectiveness of ixekizumab among patients with active psoriatic arthritis treated with secukinumab and who have had an inadequate clinical response.

## Methods

We conducted a retrospective study of patients being treated at ARTUS Health Centre in Vancouver, BC, ≥ 18 years of age with active psoriatic arthritis who, despite treatment with secukinumab 300 mg subcutaneous dosing monthly, had been switched to ixekizumab. These patients were identified by searching our electronic medical records for all patients treated with both secukinumab and ixekizumab. Patients’ physical exam including tender joint count (TJC), swollen joint count (SJC), psoriasis, axial involvement, enthesitis, and dactylitis while being treated with secukinumab was collected from chart notes prior to and after switching to ixekizumab. Duration of disease, prior DMARD therapy, concomitant DMARD therapy, primary vs secondary failure to secukinumab, and duration of ixekizumab therapy were recorded.

Tender and swollen joint count was based on a 66/68 assessment, axial involvement (confirmed by a patient’s rheumatologist with X-ray or MRI evidence of sacroiliitis) was measured by BASDAI, presence of enthesitis was measured using the SPARCC enthesitis score, and dactylitis was measured as present/absent for each finger or toe digit. Psoriasis was measured as mild, moderate, or severe based on a BSA < 3%, 3-10%, or > 10%, respectively. All patients were jointly assessed by a rheumatologist and dermatologist in a combined psoriatic arthritis clinic. Pre- and post-switch CRP was recorded when available.

## Results

Ten patients who had switched from secukinumab to ixekizumab were identified, but only eight were included in the final analysis. One patient did not return for follow-up, and another was excluded due to no formal TJC and SJC done prior to switching to ixekizumab. Table 1 summarizes characteristics and demographics of these patients.

**Table 1.** Patient Demographics and Characteristics.

Characteristic	Mean	Standard Deviation
Age (years)	51	10.3
Sex ratio (M/F)	2 males, 6 females	n/a
Psoriatic arthritis disease duration (years)	17	9.3
Average number of previously failed biologics	2.3	0.9
Average number of weeks previously treated with secukinumab	83	40.7

**Figure 1.**
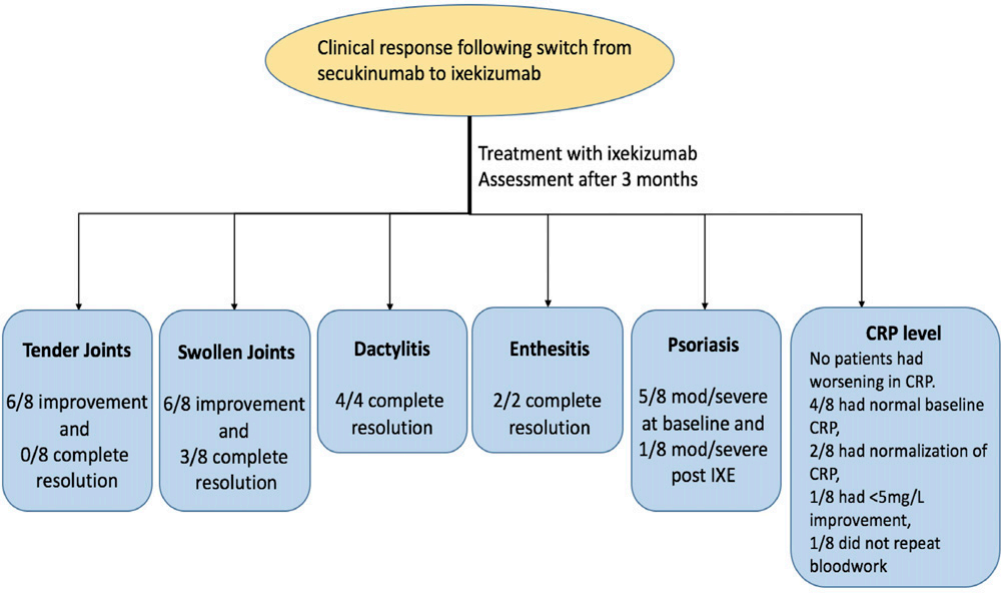
Clinical response following switch from secukinumab to ixekizumab: The figure shows response in the 5 GRAPPA domains of PsA and the CRP response.

The results of our investigations are summarized in Figure 1. Six of 8 secukinumab non-responders had previously failed TNF inhibitor therapy. Of the 8 secukinumab non-responders, 6/8 had improvement in TJC, 6/8 had improvement in SJC with 3 having complete resolution, 4/4 had complete resolution in dactylitis, 2/2 had complete resolution in enthesitis, 1/1 had improvement in BASDAI, 6/6 had improvement in psoriasis with 1 having complete clearance, and 2/6 had > 5 improvement in their CRP level. 3/8 patients were primary non-responders to secukinumab and 1 of these 3 patients had good response to ixekizumab. After 12 weeks of treatment, 6/8 patients were satisfied with their treatment response. Results of individual patients are summarized in Table 2.

**Table 2.** Patients’ Information and Changes in Their Status that Occurred after Being on Ixekizumab for At Least 3 Months.

Patient ID	Sex	Age	Duration of psoriatic arthritis (yrs)	Previously failed DMARDs and biologics	Duration of being on secukinumab	Primary vs secondary failure of secukinumab	Duration of benign ixekizumab (wk)	Concomitant DMARDs and biologics	TJC	SJC	Dactylitis	Enthesitis	Axial (BASDAJ)	Psoriasis	CRP (mg/L)
**1**	F	53	21	Methotrexate sulfasalazine secukinumab	108	Secondary	100	None	Pre: 6 Post: 1	Pre: 5 Post: 0	Pre: 2 Post: 0	Pre: 0 Post: 0		Pre: severe Post: mild	Pre: 8.1 Post: 7.2
**2**	M	45	5	Sulfasalazine leflunomide secukinumab	52	Primary	40	None	Pre: 6 Post: 2	Pre: 1 Post: 0	Pre: 1 Post: 0	Pre: 0 Post: 0	Pre: 4.4 Post: 6.7	Pre: severe Post: moderate	Pre: 1.3 Post: 1.1
**3**	F	43	22	Methotrexate sulfasalazine etanercept adalimumab ustekinumab secukinumab	64	Secondary	40	Etanercept 25 mg q2weeks	Pre: 13 Post: 5	Pre: 5 Post: 1	Pre: 0 Post: 0	Pre: 1 Post: 0		Pre: moderate Post: mild	Pre: 3.2 Post: 0.6
**4**	F	69	3	Sulfasalazine methotrexate etanercept secukinumab	52	Secondary	44	Leflunomide 10 mg	Pre: 7 Post: 3	Pre: 6 Post: 3	Pre: 2 Post: 0	Pre: 0 Post: 0		Pre: clear Post: clear	Pre: < 5 Post:< 5
**5**	F	53	21	Methotrexate leflunomide etanercept adalimumab secukinumab	156	Secondary	52	None	Pre: 26 Post: 6	Pre: 9 Post: 0	Pre: 4 Post: 0	Pre: 2 Post: 0		Pre: moderate Post: mild	Pre: 50.4 Post: n/a
**6**	M	35	21	Methotrexate sulfasalazine adalimumab secukinumab	52	Primary	52	None	Pre: 2 Post: 3	Pre: 0 Post: 1	Pre: 0 Post: 0	Pre: 0 Post: 0		Pre: severe Post: mild	Pre: 29.4 Post: 2.4
**7**	F	56	30	Methotrexate sulfasalazine apremilast secukinumab	56	Primary	52	None	Pre: 7 Post: 2	Pre: 4 Post: 2	Pre: 0 Post: 0	Pre: 0 Post: 0		Pre: mild Post: Clear	Pre: 0.8 Post: 0.5
**8**	F	57	12	Methotrexate leflunomide infliximab adalimumab secukinumab	124	Secondary	40	None	Pre: 2 Post: 6	Pre: 2 Post: 2	Pre: 0 Post: 0	Pre: 0 Post: 0		Pre: clear Post: clear	Pre: 10.7 Post: 4.1

## Discussion

This brief report presents the treatment response of 8 patients with active psoriatic arthritis who were switched from secukinumab 300 mg monthly to ixekizumab 80 mg every 4 weeks due to ongoing disease activity. A number of case series have reported improvement in cutaneous psoriasis when switching from secukinumab to ixekizumab.^5,6^ To date, there have been no studies reporting the efficacy of ixekizumab in patients with active psoriatic arthritis, and axial spondyloarthritis, and a prior inadequate response to secukinumab.

The majority of patients had failed a TNF inhibitor prior to treatment with secukinumab and were initiated at a dose of 300 mg monthly. The three patients whose first biologic was secukinumab were initiated at a dose of 150 mg monthly which was increased to 300 mg due to ongoing inflammatory arthritis.

Most patients had improvement in multiple clinical domains including tender joint count, swollen joint count, dactylitis, enthesitis, spondylitis, psoriasis, and CRP after 12 weeks of therapy with ixekizumab. Two patients were switched to a different biologic agent. This suggests that patients with active psoriatic arthritis despite therapy with secukinumab may have a clinical response following a switch to another IL17 agent. On the other hand, 3/8 patients had primary failure to secukinumab and only 1 of these 3 had adequate response to ixekizumab. This suggests that patients with secondary failure to secukinumab may have a high chance of response to ixekizumab, whereas this chance may be lower in patients with primary failure.

We did not collect patient pain VAS, patient global VAS, or Health Assessment Questionnaire Disability Index (HAQ-DI) before and after switching to ixekizumab. As a result, we were not able to determine which patients would have achieved minimal disease activity. Most patients had near-complete resolution of objective measures of disease activity such as SJC, dactylitis, psoriasis, and CRP, but only one patient was able to achieve ≤ 1 TJC, SJC, resolution of enthesitis, dactylitis, and < 3% BSA of psoriasis. Furthermore, one patient required concomitant treatment with etanercept to control disease activity.

Future real-world studies with a larger sample size are required to confirm the findings of this study. In particular, studies that focus on dactylitis and enthesitis will be able to shed more light on this subject, as a minority of patients in our study had activity in these domains. Whether there is a difference between patients with primary vs secondary failure should be further explored as well. Finally, this study did not assess whether patients would have a clinical response switching from ixekizumab to secukinumab.
